# The risk of hospitalization associated with foehn winds and temperature in the mountainous region of Switzerland

**DOI:** 10.1097/EE9.0000000000000418

**Published:** 2025-09-17

**Authors:** Tino Schneidewind, Sujung Lee, Ana Maria Vicedo-Cabrera, Apolline Saucy

**Affiliations:** a Institute for Social and Preventive Medicine, University of Bern, Bern, Switzerland; b Oeschger Centre for Climate Change Research, University of Bern, Bern, Switzerland

**Keywords:** Foehn wind, Health, Hospitalization, Heat, Weather, Climate change

## Abstract

**Background:**

Foehn winds are intense warm winds, common in mountain regions, but their health impacts and potential to exacerbate existing heat-related risks remain poorly understood. This is particularly concerning for rural areas where older, heat-vulnerable individuals live.

**Objective:**

We investigated the independent and combined association of foehn winds and temperature with cause-specific emergency hospitalizations in Switzerland.

**Methods:**

We analyzed daily temperature, foehn winds intensity, and cause-specific hospitalizations near eight foehn wind-observing meteorological stations in Switzerland (1998–2019). We performed a case-time series analysis to examine the association between foehn winds intensity and hospitalization risk with and without temperature adjustment, and whether foehn winds amplify cold and heat-related hospitalizations with an interaction term between foehn winds and temperature.

**Results:**

Foehn winds intensity showed small and no consistent association with hospitalizations in temperature-adjusted (0.4% [95% confidence interval: −1.1%, 1.8%] per 6 hours increase in daily full foehn winds intensity) and nonadjusted models (0.8% [−0.5%, 2.3%]). However, foehn winds may amplify heat-related hospitalization risk with a 14% (−3%, 33%) increase in risk at the 99th temperature percentile (vs. temperature of minimum risk) on foehn days, compared with −2% (−8%, 6%) on non-foehn days. The association was larger for females, older adults, and for hospitalizations due to respiratory and mental health causes.

**Conclusions:**

While foehn winds did not directly impact hospitalizations, they may contribute to an amplification of heat-related health risks, especially for females and older adults. Further research is needed to assess their effects in other regions, climates, and vulnerable populations.

**What this study adds**

Foehn winds are strong, warm winds common in mountainous regions, yet their health impacts remain poorly understood. Given the increasing frequency of extreme weather events and their consequences for public health, we investigated how foehn winds interact with heat to influence hospitalization risks. Using over 2 decades of hospitalization and meteorological data from Switzerland, we applied a case-time series approach to assess the independent and combined risk of foehn winds and heat on daily cause-specific hospital admissions.

Our results indicate that while foehn winds alone do not significantly impact hospitalization rates, they amplify the risk of heat-related hospitalizations, particularly among older adults, females, and individuals with respiratory or mental health outcomes. To our knowledge, this is the first assessment of its kind, providing novel knowledge on the independent and interactive risks of foehn winds and heat on hospitalizations. It is important to note that while foehn winds are mostly observed in mountainous regions, these areas could be seen as highly vulnerable since many older adults live there. With climate change expected to intensify both heat waves and atmospheric dynamics, understanding these interactions is critical for public health preparedness. Our findings underscore the need for tailored adaptation strategies and enhanced heat warning systems that account for the modifying effects of foehn winds.

## Introduction

In the era of climate change, rising global temperatures and more frequent extreme heat waves pose a growing threat to human health.^1,2^ Local meteorological conditions, including foehn winds, may influence the extent of this threat.^3^ First described in the Alps, foehn winds are a common meteorological phenomenon and occur when strong winds sweep over mountain ranges, carrying warm, dry air.^4^ Similar events occur in the Rocky Mountains^5^ and other mountainous regions worldwide, sometimes known under different names (*Santa-Ana-Wind* in California, USA, *Chinook* in the Rocky Mountains, *Puelche* in Chile, and *Chanduy* in Ecuador). Although these areas are typically not densely inhabited, they bring together a high proportion of older adults who are highly vulnerable to heat and extreme weather events. Climate change may intensify these events.^6,7^

**Figure 1.**
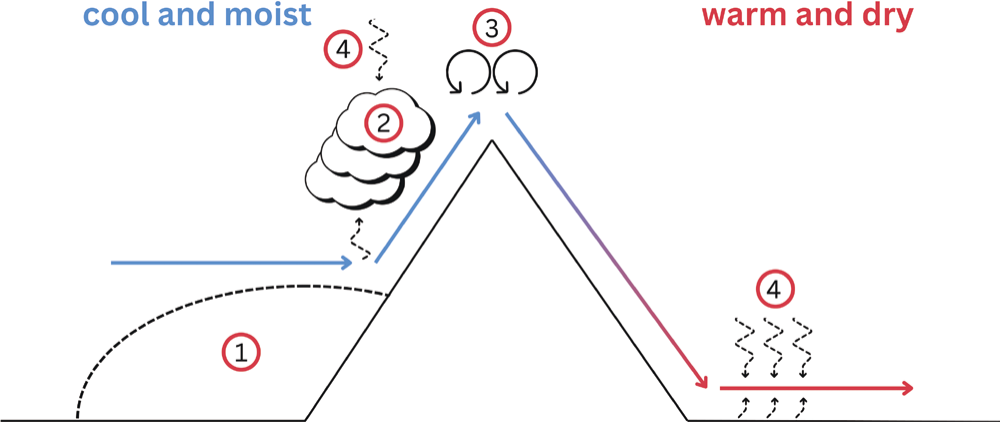
Processes that lead to foehn wind warming in the lee of mountains: (1) isentropic drawdown due to near-surface blocking; (2) latent heat release; (3) mechanical mixing; and (4) radiative heating (adapted from Elvidge and Renfrew^8^).

Foehn winds warm the air on the mountains’ downslope^8^ (Figure 1) and can reach speeds up to 270 kph.^9^ They are typically associated with low cloud cover and can last from minutes to days.^4,10^ Their warming effect results from air flowing over a mountain ridge, with factors such as near-surface air blocking,^11^ latent heat release,^10^ mechanical mixing in high altitudes,^12^ and radiative heating,^5^ contributing to the temperature increase. Foehn winds weaken when they move away from the mountains^13^ and can impact entire valleys or remain localized.^14^ While their effects are limited to alpine populations living in these areas, they can influence agriculture, ecosystems, the risks of floods and avalanches, and wildfire spread.^8,15^

Evidence on the health risks associated with foehn winds remains scattered and inconsistent, partly due to the complexity of this phenomenon involving sudden and simultaneous changes in temperature, wind speed, and humidity. In Switzerland, foehn winds have been linked with fatigue, headaches, and dizziness.^16^ Recent evidence suggests an increased risk for mental health hospitalization due to foehn wind exposure,^17^ but no association was reported for acute coronary syndromes in Switzerland.^18^ In Canada, foehn winds have been associated with an increased risk of migraines,^19^ while studies in Poland did not find any association between foehn winds and cardiovascular hospitalizations^20^ and suicide risk.^21^ A study from Germany also reported an association between foehn winds and trauma-related hospitalizations.^22^ To the best of our knowledge, no study has investigated the hospitalization risk associated with foehn winds for all-cause hospitalizations and among different age groups, nor compared their impact across different hospitalization causes. Only one study investigated the role of foehn winds on different heat-related hospitalization causes in the US and reported that foehn winds did not alter the effect of extreme heat on hospitalizations.^23^

While the effect of foehn winds on health remains inconclusive, the health impacts of heat and cold have been studied extensively. Heat and cold lead to an increasing risk of mortality^3,24–26^ and morbidity,^27–29^ and older adults are among the most affected.^30,31^ Differences between males and females are contradictory, with some studies reporting males to face greater risk,^32,33^ while others suggest females are more threatened,^34–36^ and others reporting no difference at all.^17,29^ A higher risk of heat-related hospital admission was found for respiratory,^29^ cardiovascular,^27,37^ and psychological^17,38^ causes. Cold temperatures have been associated with an increased risk for cardiovascular and respiratory causes of hospitalization.^28,39,40^ As both heat and foehn winds are expected to increase with climate change, it is particularly urgent to clarify the effects of foehn winds on health and heat-related hospital admissions across vulnerability groups. With rising global temperatures, understanding the interaction between foehn winds and heat is vital to assess their future health impacts.

This study aims to investigate the risk of hospitalization associated with foehn winds in Switzerland. We explore the effect they have on all-cause hospitalizations by sex, age, and cause of hospitalization to address the following research questions:

(1) Do foehn winds increase the risk of hospitalization, and is this association independent from temperature?

(2) Do foehn winds increase the risk of hospitalization associated with extreme temperatures?

(3) Are certain subpopulations more vulnerable to both effects than others?

To answer these questions, we performed an epidemiological investigation using data on emergency hospitalizations and weather in high-risk areas in Switzerland. We apply a case-time series design with distributed nonlinear lagged models—the state-of-the-art methods in climate epidemiology—to accurately estimate the nonlinear and delayed associations.

## Methods

### Study population and hospitalization data

**Figure 2.**
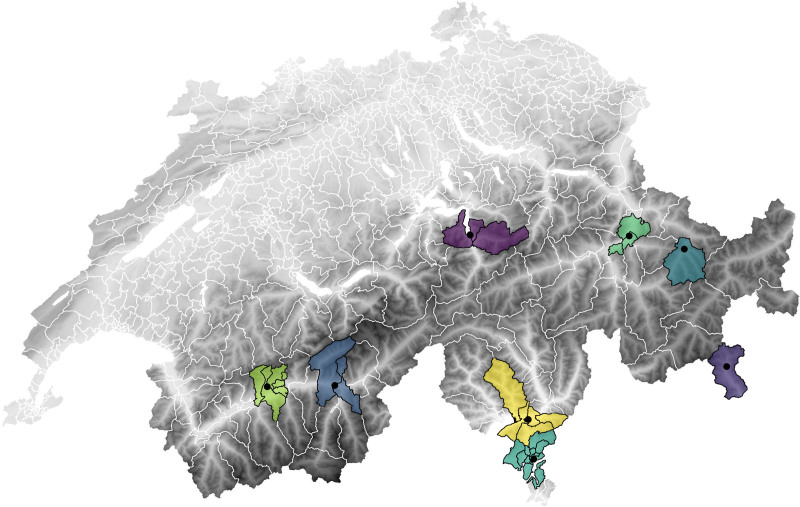
Elevation map of Switzerland with the included foehn wind-measuring meteorological stations displayed as black dots and their Medstat regions in color around them. The shading indicates elevation from 0 m above sea level (white) to 4500 m above sea level (black).

We collected daily emergency hospital admissions from populations residing around eight meteorological measurement stations that frequently observe foehn winds from the Swiss Federal Office for Statistics (Figure 2 and sTable 1; https://links.lww.com/EE/A366).^41^ Hospitalization data were available at the Medstat area level, which represents administrative health areas of variable size. We included all Medstat regions within an 8-km radius from the meteorological stations, which covered enough hospitalizations to detect an association but also excluded noise from nonaffected populations outside the foehn wind-observing valley and prevented overlapping buffers. Larger buffer sizes would have included valleys that are potentially differently affected to the foehn wind-observing valley (sTable 2 and sFigure 1; https://links.lww.com/EE/A366). Sensitivity analysis was performed on the influence of the buffer size. Specifically, Medstat regions were assigned to a station if the population density-weighted centroid of the region fell inside the station’s buffer (sTable 1; https://links.lww.com/EE/A366). The gridded population density data were provided by the Columbia Climate School Centre for Integrated Earth System Information from Columbia University.^42^

We aggregated all emergency admission by sex, age group (<65 years; >64 years), and hospitalization cause (infectious and parasitic diseases [International Classification of Diseases (ICD) code: A00-B99]; mental and behavioral disorders [ICD code: F00-F99]; diseases of the circulatory system [ICD code: I00-I99], respiratory diseases [ICD code: J00-J99]; and genitourinary diseases [ICD code: N00-N99]). We aggregated the daily Medstat data into a unique daily series for each station. We restricted our analyses to hospitalization data between January 1998 and December 2019. The start date of the series in each cluster was defined based on when data of foehn winds started to be collected (Table 1). The distribution of the hospitalization data is summarized across disease groups, stations, and over time in Table 1 and additional information in the Supplementary Material (sTable 3 and sFigures 2 and 3; https://links.lww.com/EE/A366). In 2008, the Medstat regions were redefined in their spatial extent and distribution by the Federal Office for Statistics. We performed sensitivity analysis to investigate whether the redefinition of the Medstat regions had an influence on the foehn wind-hospitalization association and on the interaction between temperature and foehn winds.

**Table 1.** Descriptive statistics for temperature, foehn wind, and hospitalizations

Area	Start date^a^	Years	Temperature^b^	Foehn wind^c^	%-Foehn wind^d^	%-bin Foehn wind^e^	Hospitalizations^f^	%-Hospitalizations^g^
All stations	1998-01-01	141	9.0 (3.1)	552 (235)	100.0%	10.3%	1152 (1304)	100.0%
Altdorf	1998-01-01	22	10.5 (0.5)	483 (114)	13.6%	8.0%	594 (155)	8.0%
Chur	1998-01-01	22	10.5 (0.6)	552 (119)	15.6%	10.9%	1710 (424)	23.2%
Davos	1998-01-07	22	4.1 (0.6)	434 (120)	12.3%	8.9%	176 (110)	2.4%
Lugano	2006-10-04	14	13.1 (1.1)	440 (156)	7.9%	8.9%	4124 (1756)	35.5%
Magadino	2006-02-15	14	12.3 (0.6)	516 (115)	9.3%	9.5%	1626 (266)	14.0%
Montana	1998-01-07	22	6.4 (0.6)	530 (210)	15.0%	9.3%	897 (709)	12.1%
Poschiavo	2008-01-23	12	8.0 (0.5)	1123 (205)	17.3%	19.0%	106 (21)	0.8%
Visp	2007-11-30	13	9.4 (3.4)	541 (187)	9.0%	11.6%	492 (157)	3.9%

a All station data ends on 31 December 2019.

b Average annual mean daily temperature and standard deviation in °C.

c Average annual sums of foehn wind duration displayed as hourly full foehn wind equivalents with standard deviations.

d The ratio of the sum of foehn wind per station compared with the sum of foehn wind from all stations.

e The percentage of days with foehn wind with a binary threshold of 72 foehn wind intensity.

f Average annual sum of hospitalization with standard deviation.

g The contribution of hospitalizations of every station to the whole data set.

### Environmental exposure data

Daily mean temperature and 10-minute foehn wind data were provided by the Swiss Federal Office for Meteorology for every monitoring station. The Federal Office classifies foehn winds into 3 levels: 0 (no foehn wind); 1 (mixed foehn wind); 2 (full foehn wind). A mixed foehn wind situation occurs when only some of the criteria for foehn wind conditions are met but not all, representing weaker foehn wind conditions than observed under full foehn wind conditions. The procedure of categorizing foehn winds is documented by Dürr.^43^

We calculated daily foehn wind intensity by summing up all 10-minute foehn wind scores in each station. Previous studies categorized foehn wind conditions based on foehn wind duration thresholds or weather charts, and did not account for daily foehn wind intensity.^17,19–22^ With our approach, 24 hours of full foehn wind equals a maximum daily score of 288. A daily foehn wind score of 144 can either correspond to 12 hours of full foehn wind, 24 hours of mixed foehn wind, or a combination of both. For the interaction analysis between foehn winds and the temperature hospitalization association, we used a binary index, using a threshold of 72 daily foehn wind intensity, which corresponds to 6 hours of full foehn wind per day, or a combination of mixed and full foehn wind. The threshold value of 72 represents a quarter of the maximum daily foehn wind score of 288, and is located at the 56th percentile of the foehn wind distribution excluding 0-foehn wind days (sFigure 2; https://links.lww.com/EE/A366). This threshold is high enough to be physically feasible, accounting for prolonged exposure, yet low enough to include enough foehn wind days to capture its effect. It also makes our findings easily comparable with previous research using a similar cutoff value for foehn wind duration.^22^ As a sensitivity analysis for our foehn wind classification method, we repeated the analyses using full foehn wind scores.

### Statistical analysis

We applied a case-time series analysis used in small-area assessments.^44^ Specifically, we used a conditional quasi-Poisson regression with distributed lag linear and nonlinear models to model the relationship between hospitalization risk, foehn winds intensity, temperature, and the interactive effect of foehn wind and temperature, over the area-specific time series data. The basic model included a four-way interaction between area, year, month, and day of the week to control for time trends in each area and changes in the potential modifiers. We addressed each of the research questions using a common base model described above and using different combinations of the terms of foehn winds intensity and temperature. Specifically, to investigate the direct association between foehn winds and hospitalizations (research question 1), we built a model with foehn winds intensities unique exposure variable (i.e., without adjusting for daily temperature) (Model 1). We consider a linear exposure-response function of the cross-basis of foehn winds intensity and an integer function for the lag response with 3 lag days. Model 2 included daily mean temperature as an explanatory variable to control for a potential confounding effect on the association between foehn winds intensity and hospitalizations. We modeled the exposure-response of daily mean temperature with a natural cubic spline with two internal knots at the 50th and 90th percentiles of the temperature distribution. The 21-day lag-hospitalization curve was modeled using a natural spline with three internal knots equally spaced on a logarithmic scale to capture possible delayed effects of cold. We tested multiple cross-basis parametrizations of the exposure-response dimension of temperature and foehn winds intensity including linear and several nonlinear functions, and performed sensitivity analyses by considering different lag periods. The resulting cross-basis parametrizations of foehn winds and temperature were chosen based on the quasi-Akaike information criterion.

Second, we investigated whether the presence of foehn winds modifies the effect of extreme temperatures on daily hospitalizations (research question 2). We estimated the association between daily mean temperature and daily hospitalizations similar to Model 2 but without adjusting for foehn winds (Model 3). Finally, Model 4 further included an interaction term between the cross-basis of temperature and foehn winds (daily binary indicator) to test whether the presence or absence of foehn winds modifies the risk of hospitalizations related to extreme temperatures. We evaluated the strength and robustness of the interaction by comparing relative risk (RR) estimates for cold and heat at the 1st and the 99th percentile of the temperature distribution in the presence and absence of foehn winds and reported RRs with 95% confidence intervals (CIs) for the interaction term.

To address research question 3, all analyses were further stratified by age and sex groups, and by hospitalization cause. Analyses were performed using RStudio (R version 4.3.2) and the *dlnm* package.^45^

## Results

### Descriptive statistics

The distribution of daily mean temperature, foehn winds variables, and hospitalizations are summarized in Table 1. The temporal extent of foehn wind-hospitalization data sets ranged between 12 and 22 years, due to different start dates across monitoring stations. The contribution of each station to total foehn wind counts ranged between 9% and 17.3%. The individual contribution of each station to total hospitalization counts ranged between 0.8% and 35.5%. A few days were excluded from the analyses due to missing values (0.6% of all days).

Daily hospitalizations included 54% males and 46% females (sTable 3; https://links.lww.com/EE/A366). Age groups were evenly spread across all stations, with 48% under 65 years, and 52% 65 years and older. The most common causes for hospitalization were cardiovascular diseases, followed by respiratory diseases. Other causes included infectious, genitourinary, and mental health diseases, and their distribution was consistent across stations, except for Montana, with 27% mental hospitalizations compared to the 17% average across all stations.

The distribution of daily foehn wind, temperature, and all-cause hospitalizations varied and peaked at different times of the year (sFigure 2; https://links.lww.com/EE/A366). Foehn wind was not observed on 77% of all days. Daily average foehn wind intensity was highest in spring, weaker in autumn and winter, and weakest in summer. The average daily foehn wind intensity score was 19, corresponding to 1.6 hours of full foehn wind. Temperature followed close to a Gaussian normal distribution, with the highest temperatures observed in July and August and the lowest temperatures from December to February. Hospitalization counts were slightly lower in summer than winter with an overall annual average of 3.2 hospitalizations per day across all stations. The seasonal pattern in hospitalizations was less pronounced than the patterns of temperatures or foehn winds intensity. We found no consistent temporal trend of foehn winds intensity or temperature (sFigure 3; https://links.lww.com/EE/A366), but observed a marked increase in hospitalizations starting in 2008. This shift coincides with the Medstat regions' redefinition and the initiation of foehn wind measurements at two stations. However, this change in the series did not substantially affect the risk estimates of foehn winds on hospitalizations (sensitivity analyses splitting the data before and after 2008, sFigure 4; https://links.lww.com/EE/A366).

### Direct association between foehn wind and daily hospitalizations

The associations between foehn winds intensity and daily hospitalizations by population subgroups are summarized in Table 2. Overall, there was no consistent association between foehn winds intensity and all-cause daily hospitalizations (0.8%; 95% CI: −0.5%, 2.3%, RR increase per 6-hour full foehn equivalent increase in Model 1). No consistent associations were observed across sex, age, or cause-specific subgroups. When adjusting for daily mean temperature in Model 2, there was no change or a slight decrease in the risk of hospitalization associated with foehn wind intensity and across subgroups. The sensitivity analysis showed no meaningful difference in risk estimates of Models 1 and 2 with different buffer sizes, lag periods for foehn winds intensity, and foehn wind aggregation methods (sTables 4–6; https://links.lww.com/EE/A366).

**Table 2.** Cumulative relative risk for hospitalizations by cause for the effect of foehn winds (Model 1) and foehn winds adjusted for daily mean temperature (Model 2) across 3 days of lag for an increase of 6 hours of full foehn wind equivalent

	Model 1 RR foehn winds	Model 2 RR foehn winds (adj. temperature)
All	1.008 (0.995–1.023)	1.004 (0.989–1.018)
Male	1.007 (0.990–1.026)	1.002 (0.984–1.021)
Female	1.010 (0.990–1.029)	1.005 (0.985–1.025)
<65 years	1.004 (0.986–1.024)	0.998 (0.979–1.018)
>64 years	1.012 (0.994–1.031)	1.009 (0.990–1.028)
Circulatory	1.000 (0.978–1.022)	1.002 (0.980–1.025)
Respiratory	1.015 (0.990–1.041)	1.003 (0.978–1.030)
Infectious	1.028 (0.996–1.060)	1.030 (0.997-1.064)
Genitourinary	1.000 (0.971–1.029)	0.989 (0.959–1.019)
Mental	1.010 (0.983–1.038)	0.997 (0.969–1.026)

95% confidence intervals are given in parenthesis.

### Foehn winds as a modifier of the association between temperature and daily hospitalizations

**Figure 3.**
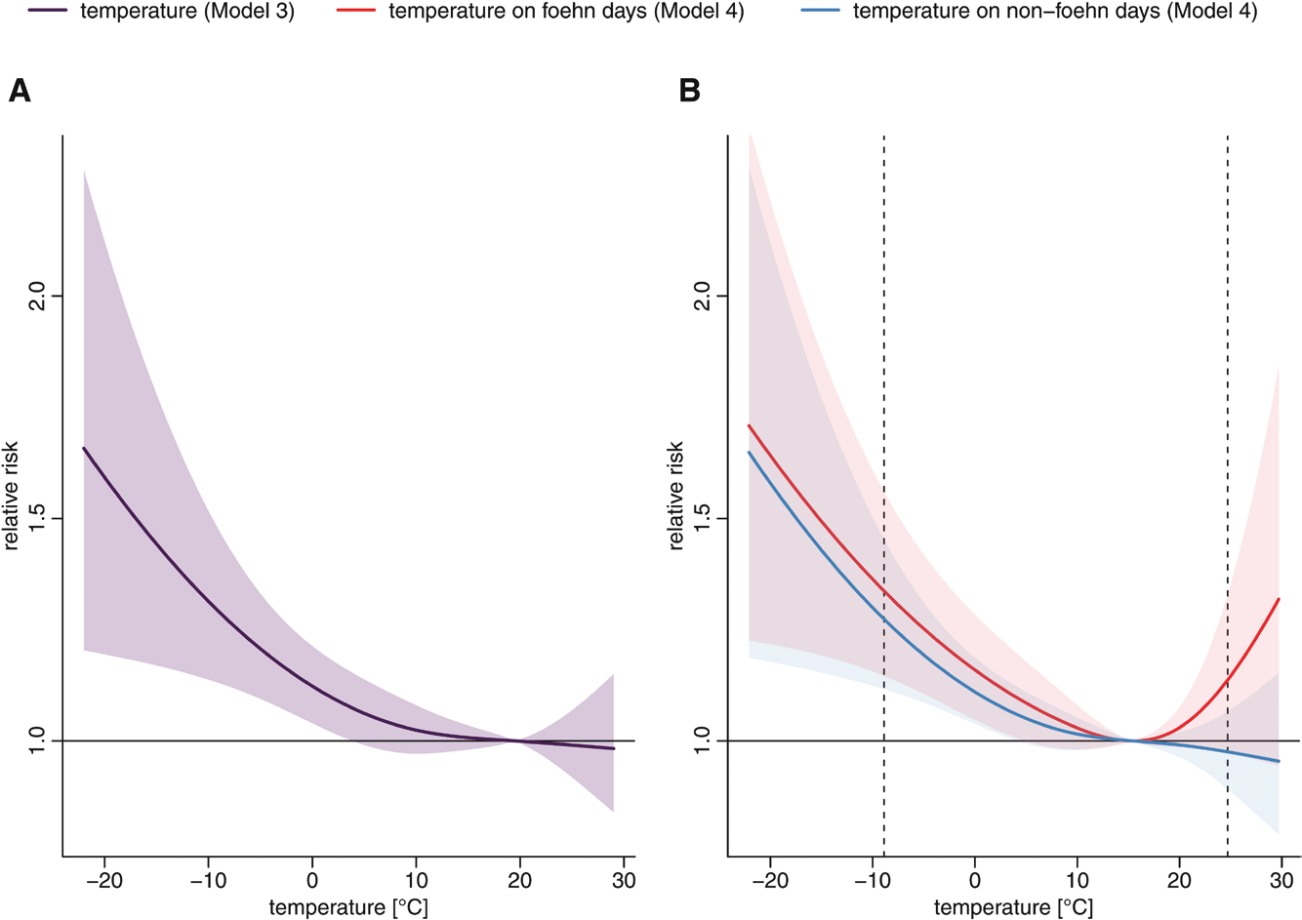
(A) Cumulative relative risk from temperature exposure (Model 3) for all-cause hospitalizations with 95% confidence interval. (B) Cumulative relative risk from temperature exposure (Model 4) for all-cause hospitalizations on foehn and non-foehn days with 95% confidence intervals. The dashed line indicates the temperature corresponding to the 1st and 99th percentile of the temperature distribution (−8.9 °C; 24.7 °C).

The results from Model 3 show a null association between heat (at 99th percentile of the temperature distribution, 24.7 °C) and hospitalization risk (−1% [95% CI: −7%, 6%]), compared with the minimum hospitalization temperature at the 91st percentile (20 °C) (i.e., the temperature value whose temperature-related risk is minimum) (Figure 3A). However, cold-related hospitalization risk increased by 29% (95% CI: 13%, 47%) at the 1st percentile of the temperature distribution (−8.9 °C) compared with the minimum hospitalization temperature.

Results from the interaction model (Model 4) indicate no evidence of difference in cold-related risk between foehn and non-foehn days (1st percentile) (Figure 3B and sFigure 5; https://links.lww.com/EE/A366). However, the heat-hospitalization risk association on foehn days increased to 14% (95% CI: −3%, 33%) at the 99th percentile of the temperature distribution (24.7°C), versus –2% (95% CI: −10%, 7%) on non-foehn days for all-cause hospitalizations, compared with the minimum hospitalization temperature of 16 °C.

**Figure 4.**
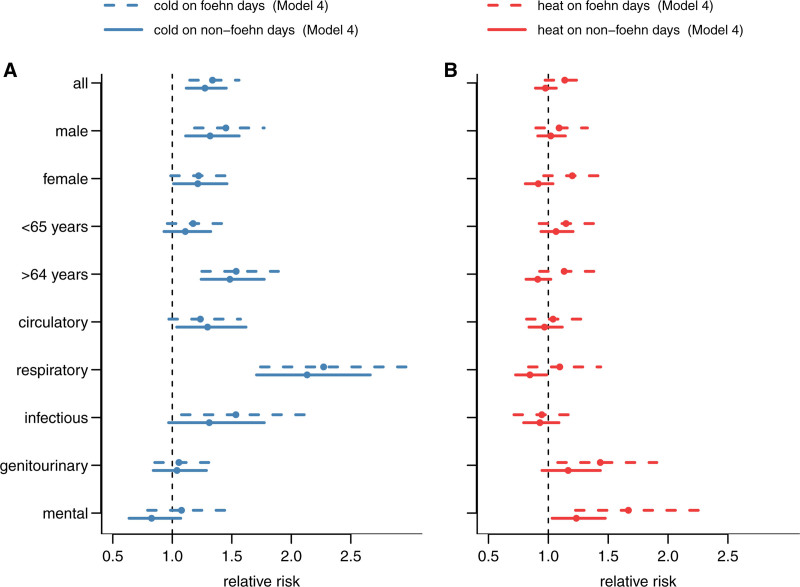
(A) Cumulative relative risk (Model 4) for subpopulations for cold temperatures (−8.9 °C, 1st percentile) with 95% confidence intervals (minimum hospitalizations temperatures and their percentiles: all [16 °C, 78th], male [16 °C, 78th], female [15 °C, 74th], 64 years and younger [14 °C, 70th], older than 64 years [16 °C, 78th], circulatory [11 °C, 57th], respiratory [19 °C, 90th], infectious [20 °C, 91st], genitourinary [0 °C, 14th], and mental [15 °C, 74th]). (B) Cumulative relative risk (Model 4) for subpopulations for heat (24.7 °C, 99th percentile) with 95% confidence intervals and the same minimum hospitalization temperatures.

The differences in heat-related risks between foehn and non-foehn days were more pronounced in specific subgroups (Figure 4B and sFigures 6 and 7; https://links.lww.com/EE/A366). Specifically, heat-related hospitalization risk in females was higher on foehn days (20% [95% CI: −4%, 50%]) than on non-foehn days (−8% [95% CI: −20%, 4%]), while risks were similar and close to null in both types of days in males. For individuals over 64 years, the risk of hospitalization increased by 22% associated with heat on foehn days to 13% (95% CI: −7%, 38%), whereas an inverse association was observed on non-foehn days. Similar risks for both foehn days and non-fohn days were also found in individuals under 65 years. Among hospitalization causes, mental-health-related hospitalization showed the highest increase in heat-related risk from 23% (95% CI: 3%, 48%) on non-foehn days to 69% (95% CI: 23%, 127%) on foehn days. For respiratory hospitalizations, which showed an inverse association with heat on non-foehn days (−13% [95% CI: −27%, −1%]), the risk became positive on foehn days (10% [95% CI: −17%, 44%]). Heat-related hospitalization risk due to genitourinary causes increased from 17% (95% CI: −5%, 44%) on non-foehn days to 43% (95% CI: 8%, 91%) on foehn days, which was, however, not significant. Circulatory and infectious disease-related hospitalizations showed only minor changes. It should be noted that from all subpopulations, we only found evidence of association between heat and hospitalization risk for mental health-related admissions on both foehn and non-foehn days, respiratory hospitalization on non-foehn days, and genitourinary hospitalizations on foehn days.

Overall, cold temperatures were associated with increased hospitalization risks on both foehn and non-foehn days for all-cause hospitalizations, males, older adults, and respiratory causes. Associations were limited to non-foehn days for females and circulatory causes, and to foehn days for infectious diseases. Cold-related hospitalization risks only differed for mental hospitalizations, increasing from −17% (95% CI: −36%, 7%) on non-foehn days to 8% (95% CI: −21%, 46%) on foehn days at the 1st percentile of the temperature distribution at (−8.9 °C). For all other subpopulations, cold-related risks were similar regardless of foehn wind conditions (Figure 4A and sFigures 6 and 7; https://links.lww.com/EE/A366).

Sensitivity analyses restricting data to 2008 onward indicated no influence of the Medstat redefinition on the interaction between foehn winds and heat-related hospitalizations, while the magnitude of the association decreased when restricting the foehn wind definition to periods with full foehn wind (sTable 7; https://links.lww.com/EE/A366).

## Discussion

To our knowledge, this is the first study to assess the association between foehn winds and cause-specific hospitalizations across sex and age groups, and their role in modifying temperature-related risks. In our study population, while no association was found between foehn winds and cause-specific emergency hospital admissions, their presence amplified heat-related hospitalization risks across multiple causes and subgroups. In contrast, similar cold-related risks were found in days with and without foehn winds, with the exception of mental-health hospitalizations with higher risk in foehn wind days, although risks were imprecise. Interestingly, a heat-hospitalization association was only found for mental and respiratory hospitalizations on non-foehn days, and for genitourinary and mental hospitalizations on foehn days. Foehn winds may increase the heat-related hospitalization risk, particularly in females, older adults, and for respiratory, mental health, and all-cause hospitalizations.

Our findings indicate that women and older adults are more vulnerable to the combined effects of foehn winds and heat, which aligns with the limited evidence existing today. Extensive evidence indicates that females and older adults face greater risk of hospitalization and mortality during heat waves.^31,46^ While we did not find an effect of foehn winds on hospitalizations in these groups, our results suggest that their hospitalization risk increases when foehn winds coincide with heat episodes. Further research is needed to confirm these findings across different populations and climate regions.

Similar to temperature-related hospitalizations, our findings by hospitalization cause suggest that foehn winds do not uniformly affect hospitalization risk but instead interact with specific health outcomes, amplifying vulnerabilities in certain subgroups. We confirm previous research showing no association between foehn winds and cardiovascular hospitalizations.^18,20^ No prior studies have examined their impact on respiratory hospitalizations, despite well-established links between heat waves and respiratory illnesses.^29^ Our findings also add nuance to research focusing on foehn winds and mental health. Mikutta et al^17^ observed a negative effect of foehn winds on the mental state of psychiatric patients. While we did not find an association between foehn winds and mental-health-related hospitalizations, our results indicate that individuals with mental health outcomes face greater hospitalization risks associated with heat and cold when foehn winds are present. These differences may result from variations in the study population and methodological approaches. However, there is robust evidence that individuals with psychological disorders are disproportionately affected by heat,^38^ and foehn winds may further exacerbate this effect. Under high and low-temperature conditions, foehn winds with their high wind speeds could act as an additional environmental stressor, amplifying mental health risks and driving higher hospitalization rates similar to those observed during heat intervals.

This study has several limitations. First, we assume equal exposure to foehn winds across all residents of the Medstat regions, even though foehn winds can occur very locally due to Switzerland’s complex topography. Second, we assume that similar foehn wind scores of different meteorological stations represent the same exposure. However, foehn wind is not a single meteorological variable but the product of multiple environmental thresholds, which have different magnitudes for each station, meaning that identical intensity scores may correspond to varying exposure intensities. As this is the first study to investigate the effects of daily foehn wind intensity, the comparability to previous studies is limited. Third, our study design may not fully capture the effect of foehn winds in colder conditions, as the lag period is potentially too short to capture harvest and long-term effects typical of low temperatures. Therefore, conclusions regarding foehn winds association in low-temperature conditions should be interpreted with caution. Given seasonal variability in ambient temperature, the effects of foehn winds may also differ across the year. While this study focused on year-round estimates for consistency and statistical power, future analyses could examine seasonal effect modification. Finally, our results should be confirmed in other mountainous regions to determine if our conclusions are unique to Switzerland’s topography and population.

## Conclusions

Although we did not find evidence that foehn winds directly impact daily hospitalizations, they may contribute to exacerbating the heat-related hospitalization risk, particularly among females and older adults, and for respiratory diseases and mental health admissions. While more frequent in mountainous regions, foehn winds are not confined to alpine areas. Climate change may intensify foehn winds and expand the annual period of foehn wind-heat interaction due to rising temperatures. Our results would justify more extensive and subpopulation-specific warning systems for foehn winds in Switzerland or adaptations of existing heat-health warning systems in mountainous areas where foehn winds are more prevalent.^24^ Further research should examine this phenomenon in other regions, with a focus on vulnerable populations, including women, children, and older individuals.

## Conflicts of interest statement

The authors declare that they have no conflicts of interest with regard to the content of this report.

## Supplementary Material

## References

[1] Vicedo-Cabrera AM Scovronick N Sera F. The burden of heat-related mortality attributable to recent human-induced climate change. Nat Clim Chang. 2021;11:492–500. 34221128 10.1038/s41558-021-01058-x PMC7611104

[2] Intergovernmental Panel on Climate Change (IPCC). Weather and Climate Extreme Events in a Changing Climate. *Climate Change 2021—The Physical Science Basis: Working Group I Contribution to the Sixth Assessment Report of the Intergovernmental Panel on Climate Change*. Cambridge University Press. 2023;1513–766.

[3] Paredes-Fortuny L Salvador C Vicedo-Cabrera AM Khodayar S. Geographical patterns in mortality impacts due to heatwaves of different characteristics in Spanish cities. Geohealth. 2024;8:e2024GH001092. 10.1029/2024GH001092 PMC11298710 39104964

[4] Burri K Hächler P Schüepp M Werner R. Der Föhnfall von April 1993. Arbeitsberichte der MeteoSchweiz. 1999.

[5] Hoinka KP. What is a foehn clearance? Bull Am Meteorol Soc. 1985;66:1123–1132.

[6] Miller NL Schlegel NJ. Climate change projected fire weather sensitivity: California Santa Ana wind occurrence. Geophys Res Lett. 2006;33:15.

[7] Hughes M Hall A Kim J. Human-induced changes in wind, temperature and relative humidity during Santa Ana events. Clim Chang. 2011;109(S1):119–132.

[8] Elvidge AD Renfrew IA. The causes of foehn warming in the lee of mountains. Bull Am Meteorol Soc. 2016;97:455–466.

[9] Sliwinska M Ciaranek D. Very strong foehn winds in the Tatra Mountains (Polish Carpathian Mountains): causes, course and consequences. Aerul *ş*i Apa: Componente ale Mediului. 2015;2015:109–116.

[10] Seibert P. South foehn studies since the ALPEX experiment. Meteorol Atmos Phys. 1990;43:91–103.

[11] Smith RB. Stratified flow over topography. In: Environmental Stratified Flows. Kluwer Academic Publishers. 2006:119–159.

[12] Scorer RS. Environmental aerodynamics. In: Cambridge Studies in Applied Econometrics. E. Horwood. 1978.

[13] Lentink HS. Extreme Foehn in Switzerland: A Climatology and the Relation to Large Scale Flow. Utrecht University. 2012.

[14] Richner H Gutermann T. Statistical analysis of foehn in Altdorf, Switzerland. In: 29th International Conference on Alpine Meteorology. 2007:457–460.

[15] Sharples JJ Mills GA McRae RHD Weber RO. Foehn-like winds and elevated fire danger conditions in Southeastern Australia. J Appl Meteorol Climatol. 2010;49:1067–1095.

[16] Berg H. Die Wirkung des Föhns auf den menschlichen Organismus. Pure Appl Geophys. 1950;17:104–111.

[17] Mikutta CA Pervilhac C Znoj H Federspiel A Müller TJ. The impact of foehn wind on mental distress among patients in a Swiss psychiatric hospital. Int J Environ Res Public Health. 2022;19:10831. 36078547 10.3390/ijerph191710831 PMC9518389

[18] Goerre S Egli C Gerber S. Impact of weather and climate on the incidence of acute coronary syndromes. Int J Cardiol. 2007;118:36–40. 16904213 10.1016/j.ijcard.2006.06.015

[19] Cooke LJ Rose MS Becker WJ. Chinook winds and migraine headache. Neurology. 2000;54:302–307. 10668687 10.1212/wnl.54.2.302

[20] Maciejczak A Guzik A Wolan-Nieroda A Wojcik M Pop T. Impact of foehn wind and related environmental variables on the incidence of cardiac events. Int J Environ Res Public Health. 2020;17:2638. 32290563 10.3390/ijerph17082638 PMC7215363

[21] Koszewska I Walawender E Baran A Zielinski J Ustrnul Z. Foehn wind as a seasonal suicide risk factor in a mountain region. Psychiatr Psychol Klin. 2019;19:48–53.

[22] Greve F Kanz KG Zyskowski M. The influence of foehn winds on the incidence of severe injuries in southern Bavaria—an analysis of the TraumaRegister DGU. BMC Musculoskelet Disord. 2020;21:568. 32825813 10.1186/s12891-020-03572-z PMC7442979

[23] Schwarz L Malig B Guzman-Morales J. The health burden of fall, winter and spring extreme heat events in Southern California and contribution of Santa Ana Winds. Environ Res Lett. 2020;15:054017.

[24] Ragettli MS Vicedo-Cabrera AM Schindler C Röösli M. Exploring the association between heat and mortality in Switzerland between 1995 and 2013. Environ Res. 2017;158:703–709. 28735231 10.1016/j.envres.2017.07.021

[25] Gasparrini A Guo Y Hashizume M. Mortality risk attributable to high and low ambient temperature: a multicountry observational study. Lancet. 2015;386:369–375. 26003380 10.1016/S0140-6736(14)62114-0 PMC4521077

[26] Pascal M Wagner V Corso M Laaidi K Ung A Beaudeau P. Heat and cold related-mortality in 18 French cities. Environ Int. 2018;121:189–198. 30216771 10.1016/j.envint.2018.08.049

[27] Michelozzi P Accetta G De Sario M; PHEWE Collaborative Group. High temperature and hospitalizations for cardiovascular and respiratory causes in 12 European cities. Am J Respir Crit Care Med. 2009;179:383–389. 19060232 10.1164/rccm.200802-217OC

[28] Fonseca-Rodríguez O Sheridan SC Lundevaller EH Schumann B. Effect of extreme hot and cold weather on cause-specific hospitalizations in Sweden: A time series analysis. Environ Res. 2021;193:110535. 33271141 10.1016/j.envres.2020.110535

[29] Zhao Q Li S Coelho MSZS. Assessment of intraseasonal variation in hospitalization associated with heat exposure in Brazil. JAMA Netw Open. 2019;2:e187901. 30735233 10.1001/jamanetworkopen.2018.7901 PMC6484586

[30] Benmarhnia T Deguen S Kaufman JS Smargiassi A. Review Article: vulnerability to heat-related mortality: a systematic review, meta-analysis, and meta-regression analysis. Epidemiology. 2015;26:781–793. 26332052 10.1097/EDE.0000000000000375

[31] Vicedo-Cabrera AM Ragettli MS Schindler C Röösli M. Excess mortality during the warm summer of 2015 in Switzerland. Swiss Med Wkly. 2016;146:w14379. 10.4414/smw.2016.14379 27922165

[32] Diaz J Linares C Tobias A. Impact of extreme temperatures on daily mortality in Madrid (Spain) among the 45–64 age-group. Int J Biometeorol. 2006;50:342–348. 16718468 10.1007/s00484-006-0033-z

[33] Bell ML O’Neill MS Ranjit N Borja-Aburto VH Cifuentes LA Gouveia NC. Vulnerability to heat-related mortality in Latin America: a case-crossover study in São Paulo, Brazil, Santiago, Chile and Mexico City, Mexico. Int J Epidemiol. 2008;37:796–804. 18511489 10.1093/ije/dyn094 PMC2734062

[34] Rainham DGC Smoyer-Tomic KE. The role of air pollution in the relationship between a heat stress index and human mortality in Toronto. Environ Res. 2003;93:9–19. 12865043 10.1016/s0013-9351(03)00060-4

[35] Hajat S Armstrong B Baccini M. Impact of high temperatures on mortality: Is there an added heat wave effect? Epidemiology. 2006;17:632–638. 17003686 10.1097/01.ede.0000239688.70829.63

[36] Vaneckova P Beggs PJ de Dear RJ McCracken KWJ. Effect of temperature on mortality during the six warmer months in Sydney, Australia, between 1993 and 2004. Environ Res. 2008;108:361–369. 18774130 10.1016/j.envres.2008.07.015

[37] Wallace RF Kriebel D Punnett L Wegman DH Amoroso PJ. Prior heat illness hospitalization and risk of early death. Environ Res. 2007;104:290–295. 17306249 10.1016/j.envres.2007.01.003

[38] Zhang Y Nitschke M Krackowizer A. Risk factors of direct heat-related hospital admissions during the 2009 heatwave in Adelaide, Australia: a matched case-control study. BMJ Open. 2016;6:e010666. 10.1136/bmjopen-2015-010666 PMC4893849 27256088

[39] Tian L Qiu H Sun S Phil M Lin H. Emergency cardiovascular hospitalization risk attributable to cold temperatures in Hong Kong. Circ Cardiovasc Qual Outcomes. 2016;9:135–142. 26933049 10.1161/CIRCOUTCOMES.115.002410

[40] Feng J Cao D Zheng D. Cold spells linked with respiratory disease hospitalization, length of hospital stay, and hospital expenses: exploring cumulative and harvesting effects. Sci Total Environ. 2023;863:160726. 36502973 10.1016/j.scitotenv.2022.160726

[41] Schweizerische Eidgenossenschaft. Bundesamt für Statistik. Medizinische Statstik der Krankenhäuser. Available at: https://www.bfs.admin.ch/bfs/de/home/statistiken/gesundheit/erhebungen/ms.html. Accessed 3 October 2024.

[42] Center For International Earth Science Information Network-CIESIN-Columbia University. Gridded Population of the World, Version 4 (GPWv4): Population Count Adjusted to Match 2015 Revision of UN WPP Country Totals, Revision 11. Palisades, NY: NASA Socioeconomic Data and Applications Center (SEDAC); 2018. Available at: https://earthdata.nasa.gov/data/catalog/sedac-ciesin-sedac-gpwv4-apct-wpp-2015-r11-4.11. Accessed 10 October 2024.

[43] Dürr B. Automatisiertes Verfahren zur Bestimmung von Föhn in Alpentälern. Arbeitsberichte der MeteoSchweiz. 2008:223.

[44] Gasparrini A. A tutorial on the case time series design for small-area analysis. BMC Med Res Methodol. 2022;22:129. 35501713 10.1186/s12874-022-01612-x PMC9063281

[45] Gasparrini A. Distributed lag linear and non-linear models in R: the package dlnm. J Stat Softw. 2011;43:1–20. PMC3191524 22003319

[46] Grize L Huss A Thommen O Schindler C Braun-Fahrländer C. Heat wave 2003 and mortality in Switzerland. Swiss Med Wkly. 2005;135:200–205. 15909237 10.4414/smw.2005.11009

