# Supplementary material for: The risk of hospitalization associated with foehn winds and temperature in the mountainous region of Switzerland
Source: Environ Epidemiol. 2025 Sep 17;9(5):e418. doi: 10.1097/EE9.0000000000000418 (PMC12445415; doi:10.1097/EE9.0000000000000418)
Supplement: Supplementary file 1 [file ee9-9-e418-s001.pdf]

Supplementary Material

sTable 1: *Abbreviation and location of meteorological measurement stations and their assigned Medstat regions.*

	Altdorf	Chur	Davos	Lugano	Magadino	Montana	Poschiavo	Visp
Abbreviation	ALT	CHU	DAV	LUG	MAG	MVE	ROB	VIS
X Coordinate	46.887042	46.870622	46.812956	46.003833	46.160019	46.298772	46.347194	46.302875
Y Coordinate	8.621806	9.530814	9.843492	8.960117	8.933608	7.460761	10.062964	7.842903
Medstat regions	UR01	GR01	GR09	TI20	TI16	VS12	GR25	VS09
	UR02	GR04		TI21	TI06	VS15		VS06
	UR03	GR03		TI22	TI15	VS16		
				TI25	TI07	VS19		
				TI27	TI17	VS13		
				TI18				
				TI23				
				TI19				
				TI28				
				TI29				
				TI26				
				TI24				

sTable 2: The number of total Medstat regions per buffer radius size in km.

buffer radius (km)	number of Medstat regions
2	4
3	8
4	13

5	18
6	24
7	26
8	32
9	35
10	38
11	41
12	46
13	46
14	50
15	53

sTable 3: *Contribution in percent of different subpopulations to all-cause hospitalization counts across all stations and for every station.*

Area	all	male	female	<65 years	>64 years	circulatory	respiratory	infectious	genitourinary	mental
all stations	162458	53.9%	46.1%	47.6%	52.4%	33.9%	22.1%	12.1%	15.0%	16.9%
Altdorf	13057	55.7%	44.3%	46.9%	53.1%	40.2%	18.2%	14.8%	12.6%	14.2%
Chur	37614	54.0%	46.0%	57.5%	42.5%	31.2%	21.7%	12.9%	14.7%	19.5%
Davos	3871	51.7%	48.3%	51.9%	48.1%	33.3%	21.8%	15.5%	14.0%	15.4%
Lugano	57742	53.2%	46.8%	40.1%	59.9%	35.9%	23.1%	11.0%	16.4%	13.6%
Magadino	22771	54.5%	45.5%	44.6%	55.4%	33.8%	23.7%	11.8%	16.3%	14.4%
Montana	19736	54.1%	45.9%	52.1%	47.9%	30.8%	20.3%	9.9%	11.9%	27.1%
Poschiavo	1274	53.8%	46.2%	40.7%	59.3%	34.8%	23.1%	8.7%	14.4%	19.0%
Visp	6393	55.0%	45.0%	53.9%	46.1%	29.3%	23.2%	17.4%	15.9%	14.1%

sTable 4: *Results (relative risks) for the sensitivity analysis of different buffer sizes for all-cause hospitalizations with 95% confidence intervals.*

Buffer radius	Model 1	Model 2
5 km	1.002 [0.985-1.019]	0.998 [0.981-1.016]
10 km	1.007 [0.995-1.020]	1.004 [0.991-1.017]

sTable 5: *Results (relative risks) for the sensitivity analysis on different lag periods for all cause hospitalizations with 95% confidence intervals.*

Lag	Model 1	Model 2
1 days	1.006 [0.995-1.017]	0.001 [0.989-1.012]
5 days	0.999 [0.983-1.016]	0.996 [0.979-1.013]
10 days	1.003 [0.980-1-027]	1.006 [0.982-1.030]
15 days	0.992 [0.962-1.023]	1.001 [0.970-1.033]

sTable 6: *Results (relative risks) for the sensitivity analysis on the influence of the foehn wind aggregation method (only full foehn wind) on the association between foehn wind exposure with accounting for temperature confounding (Model 2). The left column represents the baseline risk shown in Table 2.*

	Model 2 baseline	Model 2 only full foehn wind
All	1.004 [0.989-1.018]	1.001 [0.987-1.016]
Male	1.002 [0.984-1.021]	1.000 [0.981-1.019]
Female	1.005 [0.985-1.025]	1.003 [0.983-1.024]
<65 years	0.998 [0.979-1.018]	0.988 [0.968-1.008]
>64 years	1.009 [0.990-1.028]	1.013 [0.994-1.033]
Circulatory	1.002 [0.980-1.025]	1.002 [0.979-1.025]
Respiratory	1.003 [0.978-1.030]	1.006 [0.980-1.034]
Infectious	1.030 [0.997-1.064]	1.012 [0.979-1.047]
Genitourinary	0.989 [0.959-1.019]	0.984 [0.954-1.016]
Mental	0.997 [0.969-1.026]	0.996 [0.966-1.025]

sTable 7: *Results (relative risks) for the sensitivity analysis on the influence of the observation period (1998-2019 and 2008-2019) and the foehn wind aggregation method (only full foehn wind) on the interaction between heat (24.7°C, 99th percentile) and foehn winds (Model 4) with fixed minimum hospitalization temperatures for every subpopulation. The 2 left columns represent the baseline risk shown in Figure 4b.*

Subpopulation	***1998-2019: foehn days***	***1998-2019: non-foehn days***	***2008-2019: foehn days***	**2008-2019 non-foehn days**	***Full foehn wind 1998-2019: foehn days***	***Full foehn wind 1998-2019: non-foehn days***

All	*1.137 [0.973-1.329]*	*0.976 [0.893-1.066]*	*1.167 [0.971-1.404]*	*0.939 [0.844-1.045]*	*1.018 [0.869-1.192]*	*0.956 [0.879-1.039]*
Male	*1.090 [0.896-1.326]*	*1.021 [0.912-1.142]*	*1.096 [0.865-1.389]*	*1.023 [0.894-1.172]*	*1.055 [0.861-1.293]*	*1.008 [0.905-1.123]*
Female	*1.200 [0.961-1.499]*	*0.916 [0.808-1.039]*	*1.236 [0.951-1.606]*	*0.842 [0.723-0.981]*	*0.972 [0.776-1.219]*	*0.887 [0.787-1.000]*
<65 years	*1.148 [0.924-1.426]*	*1.064 [0.939-1.206]*	*1.140 [0.878-1.481]*	*0.948 [0.811-1.109]*	*1.055 [0.843-1.320]*	*1.066 [0.946-1.202]*
>64 years	*1.132 [0.927-1.383]*	*0.911 [0.814-1.021]*	*1.195 [0.942-1.515]*	*0.934 [0.816-1.068]*	*0.993 [0.808-1.219]*	*0.880 [0.790-0.980]*
Circulatory	*1.039 [0.818-1.321]*	*0.968 [0.839-1.116]*	*1.009 [0.753-1.352]*	*0.915 [0.763-1.097]*	*0.854 [0.665-1.096]*	*0.925 [0.806-1.061]*
Respiratory	*1.096 [0.835-1.438]*	*0.846 [0.726-0.986]*	*1.259 [0.903-1.754]*	*0.812 [0.675-0.976]*	*0.953 [0.722-1.260]*	*0.799 [0.690-0.926]*
Infectious	*0.945 [0.713-1.253]*	*0.930 [0.794-1.089]*	*0.758 [0.534-1.076]*	*0.871 [0.719-1.055]*	*0.967 [0.723-1.293]*	*0.993 [0.854-1.155]*
Genitourinary	*1.434 [1.079-1.906]*	*1.166 [0.947-1.435]*	*1.903 [1.325-2.734]*	*1.377 [1.045-1.816]*	*1.517 [1.144-2.012]*	*1.191 [0.979-1.450]*
Mental	*1.669 [1.227-2.269]*	*1.234 [1.032-1.476]*	*1.636 [1.130-2.367]*	*1.115 [0.897-1.386]*	*1.371 [0.998-1.885]*	*1.167 [0.984-1.384]*

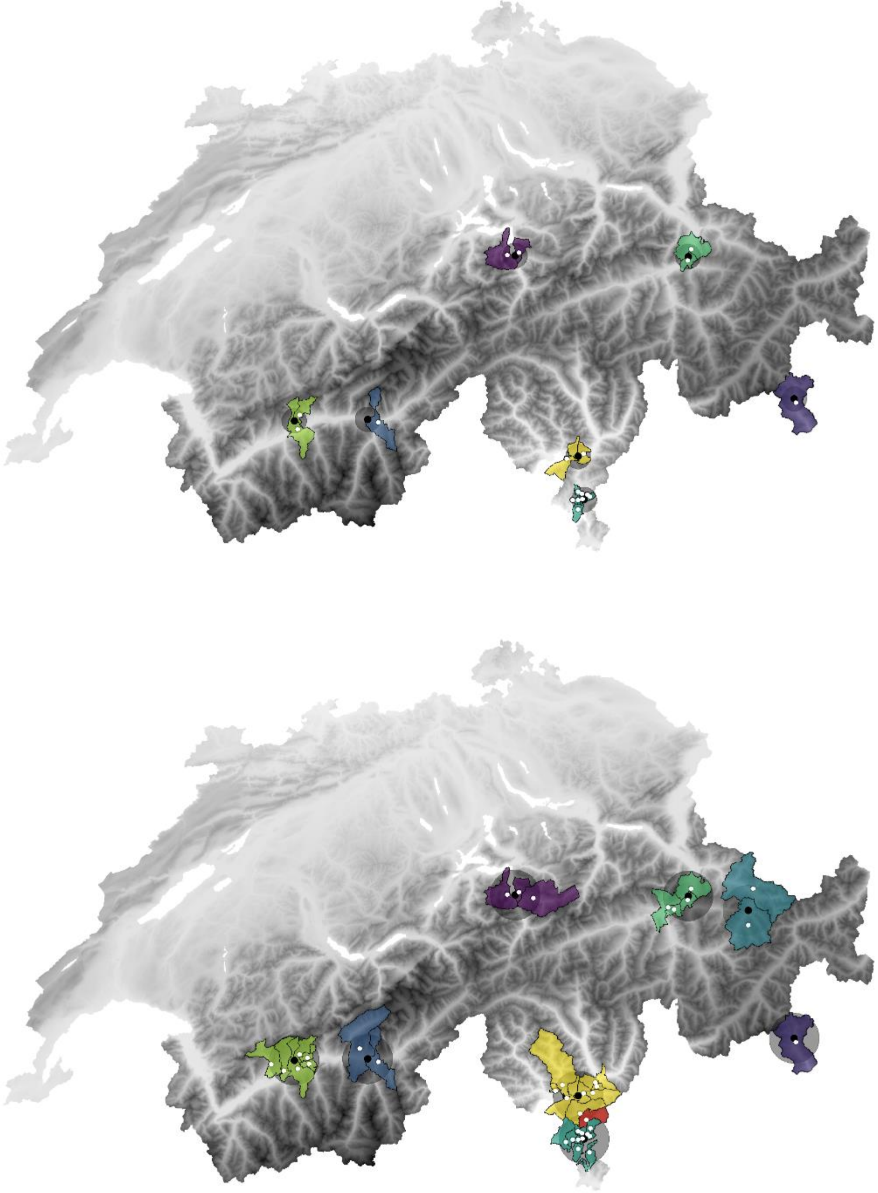

sFigure 1: *Elevation map of Switzerland with the included foehn-measuring meteorological stations displayed as black dots and their Medstat regions in color around them. Population density-weighted centroids of each selected Medstat region are shown as white dots. The red Medstat region is in the buffer radius of two meteorological stations. Top: buffer radius of 5 km. Bottom: buffer radius of 10 km.*

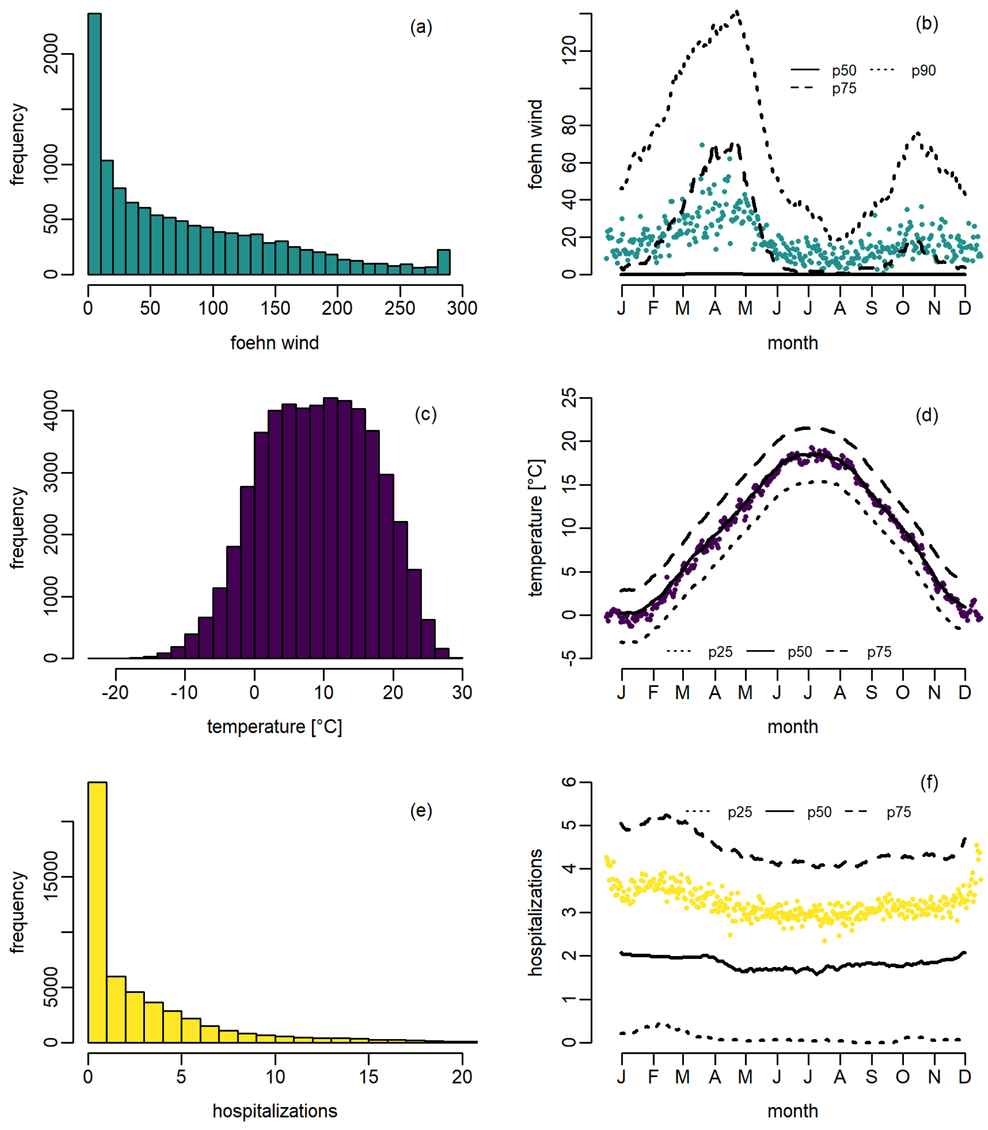

sFigure 2: *(a) daily foehn wind intensity score distribution excluding 0-foehn wind days, (b) daily mean foehn wind intensity with 30-day moving averages of the 50th, 75th, 90th percentile, (c) daily mean temperature distribution, (d) daily averages of daily mean temperature with 30-day moving averages of the 25th, 50th, 75th percentile, (e) daily all-cause hospitalization distribution, (f) daily mean all-cause hospitalizations with 30-day moving averages of the 25th, 50th, 75th percentile.*

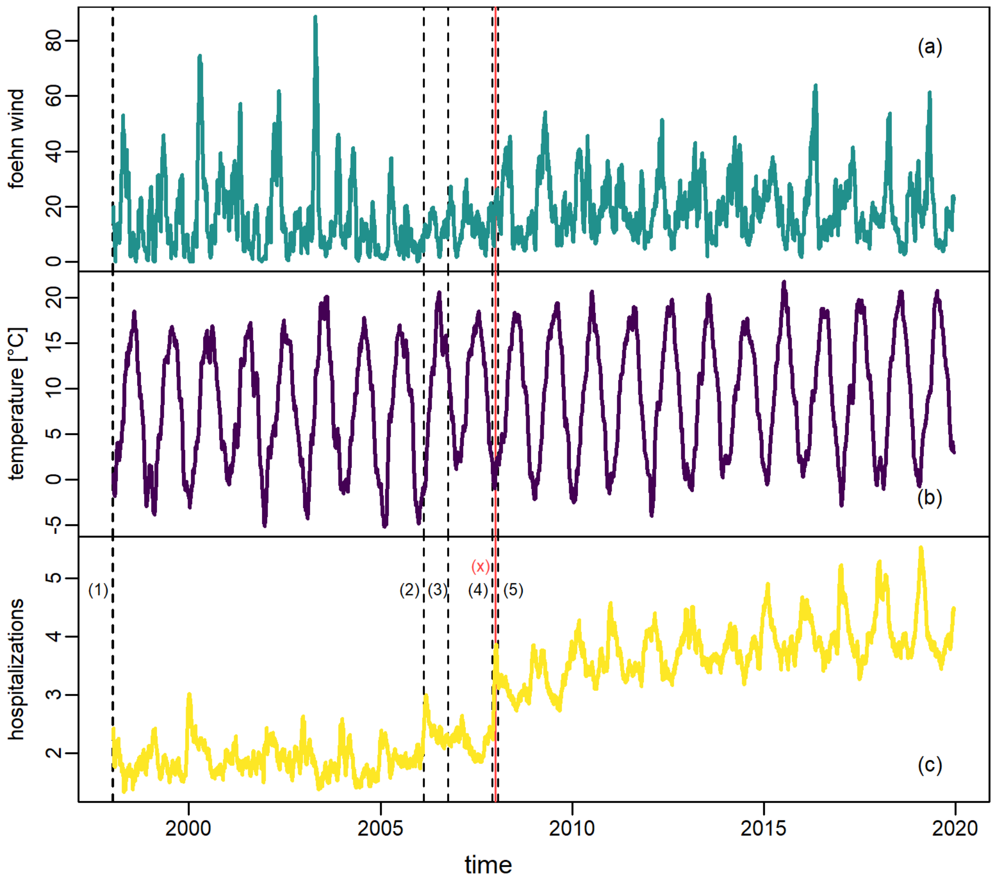

sFigure 3: *(a) 30-day moving average time series of mean daily foehn wind intensity, (b) 30-day moving average time series of average daily mean temperature, (c) 30-day moving average of mean daily all-cause hospitalization. (1) start of data from Altdorf, Chur, Davos, Montana, (2) start of data from Magadino, (3) start of data from Lugano, (4) start of data from Visp, (5) start of data from Poschiavo. (x) redefinition of the Medstat regions by the Swiss Federal Office for Statistics.*

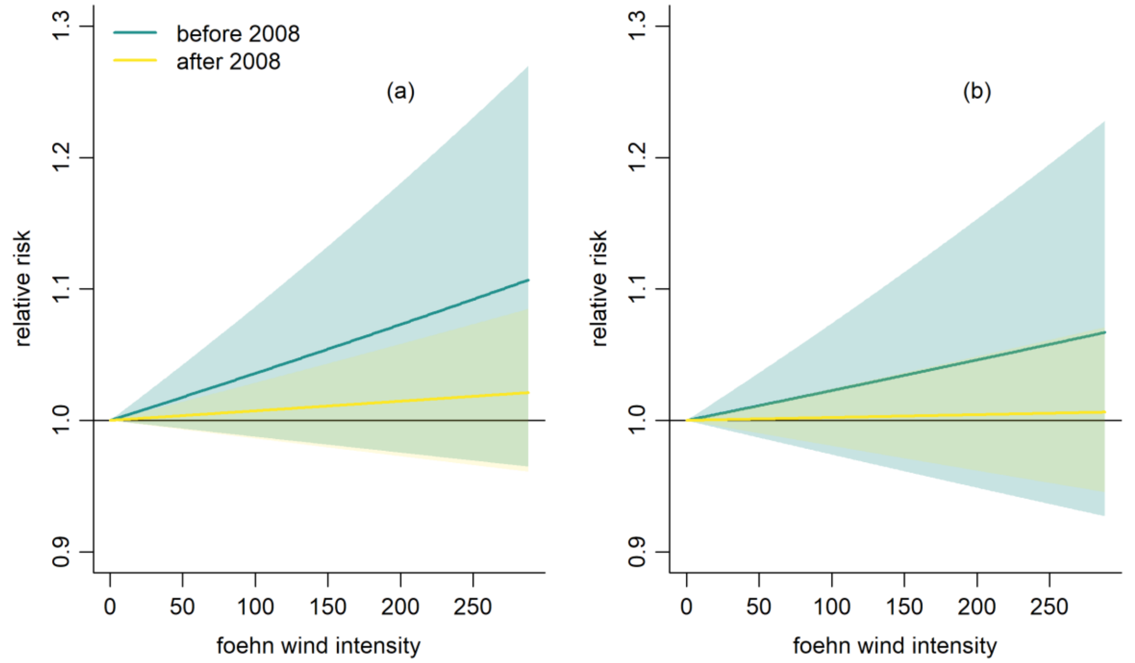

sFigure 4: *Cumulative relative risk for all-cause hospitalizations for Model 1 (a) and Model 2 (b) for data sets split in 2008.*

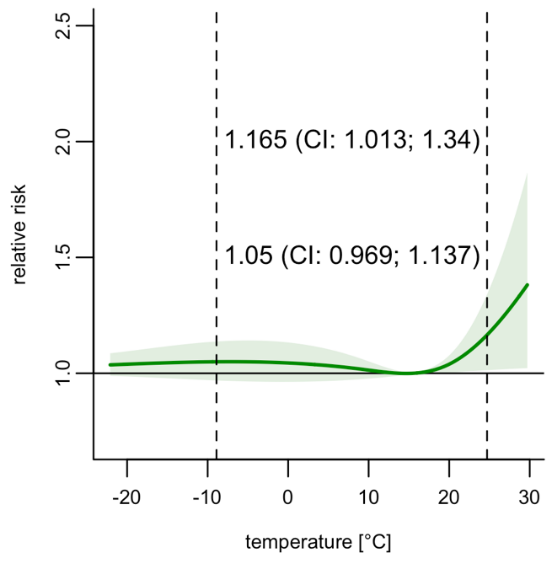

sFigure 5: *Cumulative relative risk of the interaction between foehn winds and temperature with 95% confidence interval for all-cause hospitalizations. The relative risk and the confidence interval shown inside the figure correspond to the relative risk at the exposure of -8.9 °C (1st percentile)(bottom) and 24.7°C (99th percentile)(top).*

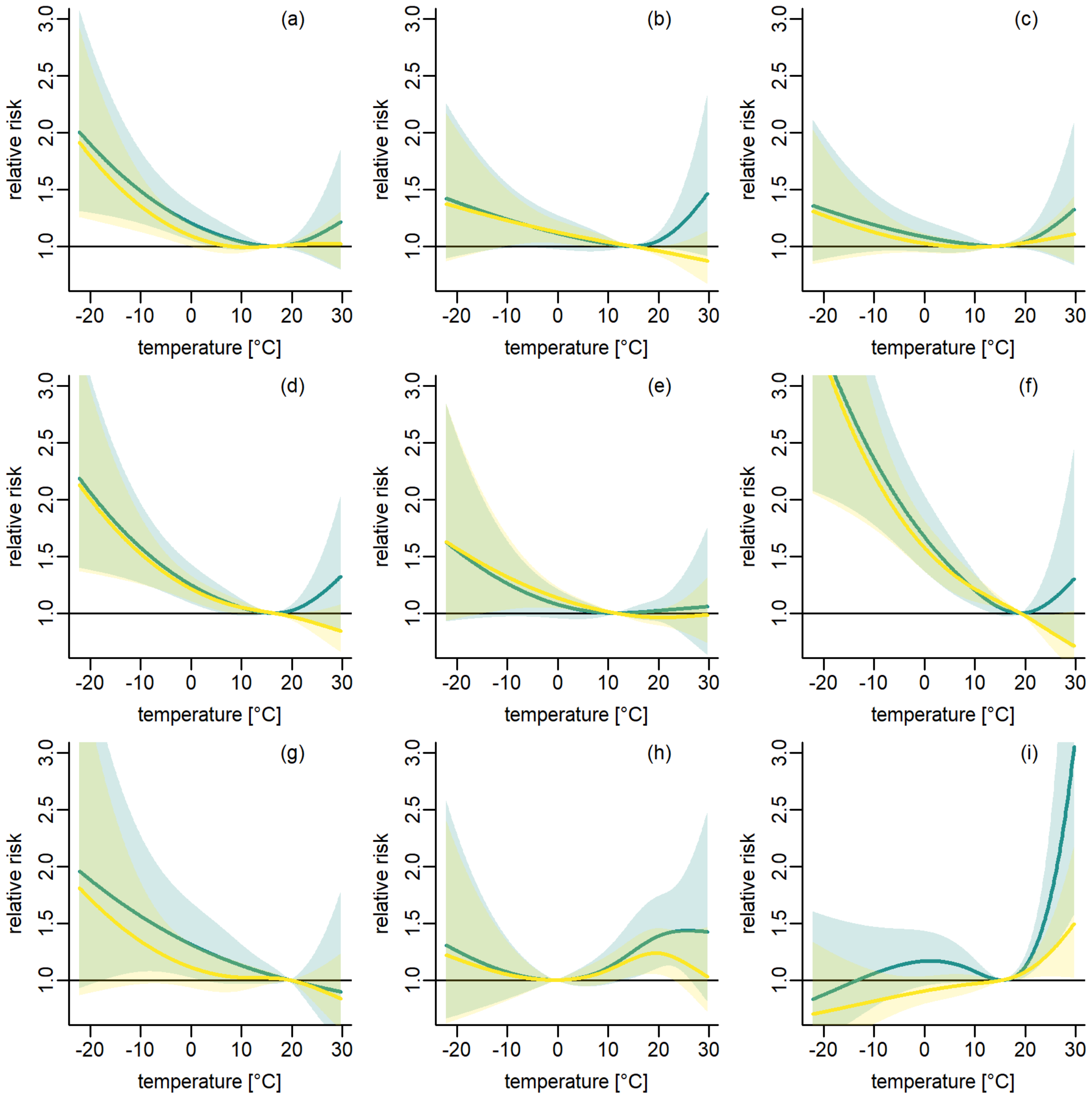

sFigure 6: Cumulative relative risk with 95% confidence intervals and minimum hospitalization temperatures with their percentile for (a) male [16°C, 78th], (b) female [15°C, 74th], (c) 64 years and younger [14°C, 70th], (d) older than 64 years [16°C, 78th], (e) circulatory [11°C, 57th], (f) respiratory [19°C, 90th], (g) infectious [20°C, 91st], (h) genitourinary [0°C, 14th], (i) mental hospitalization [15°C, 74th] with a binary foehn wind intensity threshold value of 72 which corresponds to 6 hours of full foehn wind. The green line shows the temperature hospitalization association when foehn winds were present, the yellow line when foehn winds were absent.

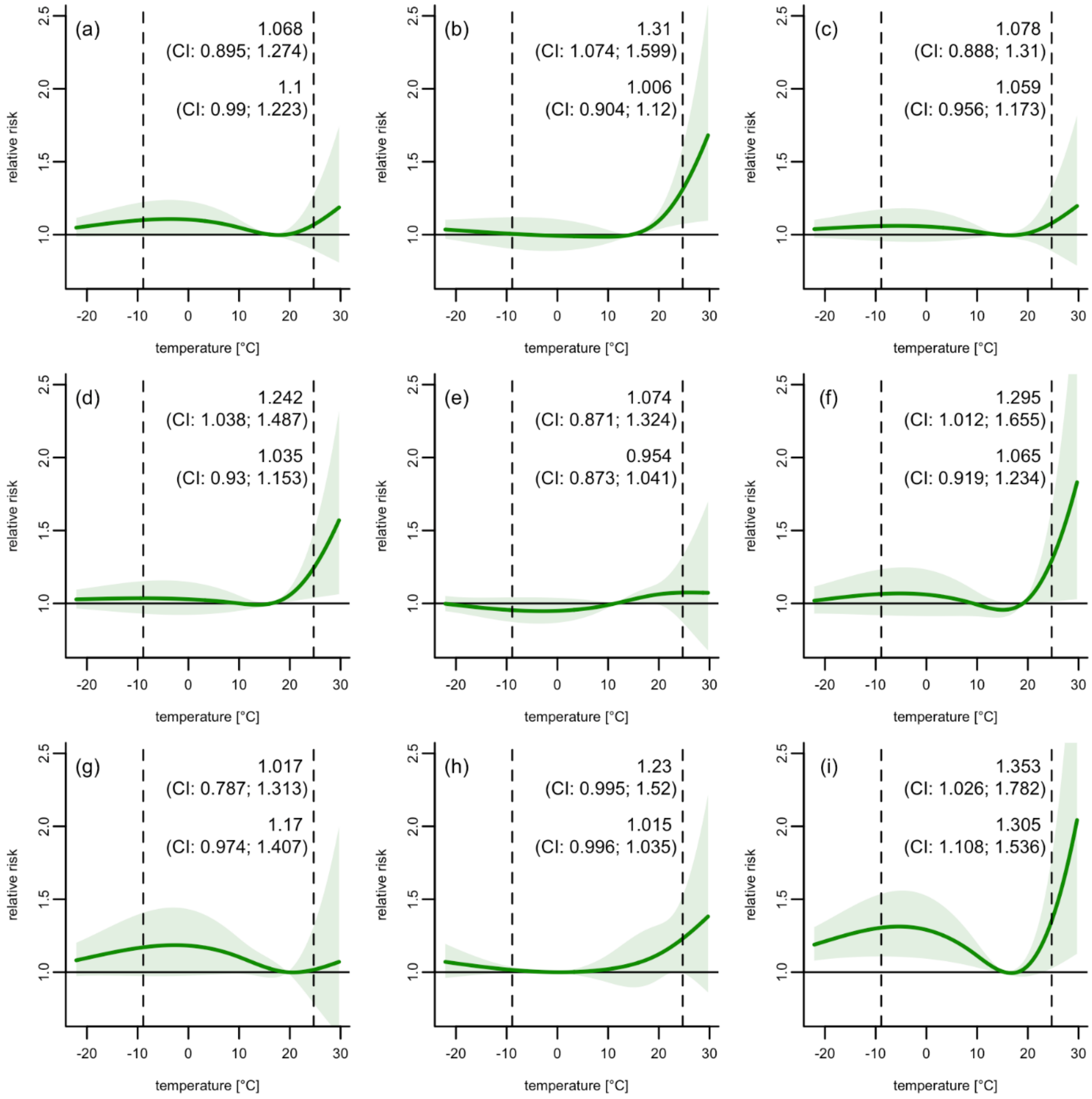

sFigure 7: *Cumulative relative risk of the interaction between foehn wind and temperature with 95% confidence interval for (a) male, (b) female, (c) 64 years and younger, (d) older than 64, (e) circulatory, (f) respiratory, (g) infectious, (h) genitourinary, (i) mental hospitalizations. The relative risk and the confidence interval shown inside each figure correspond to the relative risk at the exposure of -8.9 °C (1st percentile)(bottom) and 24.7°C (99th percentile)(top).*
